# Host Plants Identification for Adult *Agrotis ipsilon*, a Long-Distance Migratory Insect

**DOI:** 10.3390/ijms17060851

**Published:** 2016-06-02

**Authors:** Yongqiang Liu, Xiaowei Fu, Limi Mao, Zhenlong Xing, Kongming Wu

**Affiliations:** 1 State Key Laboratory for Biology of Plant Diseases and Insect Pests, Institute of Plant Protection, Chinese Academy of Agricultural Sciences, Beijing 100193, China; lyq364467268@163.com (Y.L.); fxw1983@126.com (X.F.); longtaitou100@126.com (Z.X.); 2 Nanjing Institute of Geology and Palaeontology, Chinese Academy of Sciences, Nanjing 210008, China; lmmao@nigpas.ac.cn

**Keywords:** *Agrotis ipsilon*, pollen, molecular identification, “insect-host plant” coevolution

## Abstract

In this study, we determined the host relationship of *Agrotis ipsilon* moths by identifying pollen species adhering them during their long-distance migration. Pollen carried by *A. ipsilon* moths was collected from 2012 to 2014 on a small island in the center of the Bohai Strait, which is a seasonal migration pathway of this pest species. Genomic DNA of single pollen grains was amplified by using whole genome amplification technology, and a portion of the chloroplast *rbcL* sequence was then amplified from this material. Pollen species were identified by a combination of DNA barcoding and pollen morphology. We found 28 species of pollen from 18 families on the tested moths, mainly from Angiosperm, Dicotyledoneae. From this, we were able to determine that these moths visit woody plants more than herbaceous plants that they carry more pollen in the early and late stages of the migration season, and that the amounts of pollen transportation were related to moth sex, moth body part, and plant species. In general, 31% of female and 26% of male moths were found to be carrying pollen. Amounts of pollen on the proboscis was higher for female than male moths, while the reverse was true for pollen loads on the antennae. This work provides a new approach to study the interactions between noctuid moth and their host plants. Identification of plant hosts for adult moths furthers understanding of the coevolution processes between moths and their host plants.

## 1. Introduction

Plants and their insect herbivores represent more than 50% of all known species on earth [1]. The interaction between these two ecological partners is one of the most common and consequential of ecological associations [2]. Flowers and insects co-evolved [3]. Plants depending upon insects for pollination have evolved pollen that adheres readily to the insects’ exterior [4]. The yield of many crops usually increases with the number of pollinators, meanwhile, adults of numerous insect species feed on nectar, pollen and other plant exudates that are frequently associated with flowers [3]. The first step in understanding how these ecological associations function is to determine the range of host plants used by particular herbivorous insects.

Noctuidae is the largest family of Lepidoptera, containing over 40,000 currently recognized species [5]. Noctuids are a prominent feature of terrestrial insect faunas and food webs, including many species of economic importance [6]. Within the noctuids, larvae and adults usually differ considerably in their nutritional requirements and food ecology. Often, larvae feed on the structural tissue of their host plants while adults feed primarily or exclusively on plant-provided food supplements such as nectar and pollen [7]. Many studies focus on larval host records by direct observations of herbivory in the field and laboratory feeding trials [8,9,10,11]. Unfortunately, identifying the diet of adults using these methods is challenging. Direct observation of noctuid moths is limited by their nocturnal and flight habits. In laboratory-feeding trials, insects will often feed on host plants not normally consumed in nature, resulting in an overestimation of their diet breadths [8].

Nectar is a sugar-rich plant secretion that may contain low levels of amino acids, proteins, lipids, vitamins and secondary plant compounds, as well as other organic compounds and minerals [12]. Adults of many insect species feed on nectar to meet nutritional requirements for successful reproduction. As a result of this feeding activity, adults become contaminated with pollen, and the identification of pollen species can be used to determine the insect’s host plants [3]. Although conventional pollen morphology is widely used for plant identification, in some cases, pollen of closely related plants has highly similar morphology [13,14].

Alternatively, DNA bar coding has great potential for determining the dietary compositions of organisms [15,16,17,18]. For this purpose, a partial region of plant DNA sequence needs to be amplified, and compared to a reference database such as GenBank [15,16]. Genomic DNA of single pollen grains can be amplified by using the whole genome amplification technology for identification of the source plant species [19]. Previous studies have demonstrated that such molecular markers have the potential to identify insect herbivore diets at the plant family or genus level [15,16]. Accurate host identification is affected by the completeness of the database of reference DNA sequences, reference libraries are also used [15,16,18]. Moreover, the unambiguous identification of host plants at the species-level using this approach is not yet certain [18]. Therefore, in the present study, a combined approach of DNA barcoding and pollen morphology was used to identify pollen species.

The black cutworm, *Agrotis ipsilon* (Hufnagel) (Lepidoptera: Noctuidae), is a key pest of cotton, maize, potatoes, beans, and other crops [20]. Its long-distance migration behavior has allowed this species to disperse throughout Africa, Europe, Asia, and the Americas [21,22,23]. Wu and Guo (1997) and Jiang *et al.* (2000) found that adult feeding can significantly increase the flight capacity of moths [24,25], and that migratory *A. ipsilon* were often contaminated with pollen [26]. Therefore, in this study, the pollen found adhered to *A. ipsilon* moths was identified. We also investigated the quantities of pollen attached to various parts of bodies of *A. ipsilon* moths.

## 2. Results

### 2.1. Plant Hosts Inferred from Pollen

**Figure 1:**
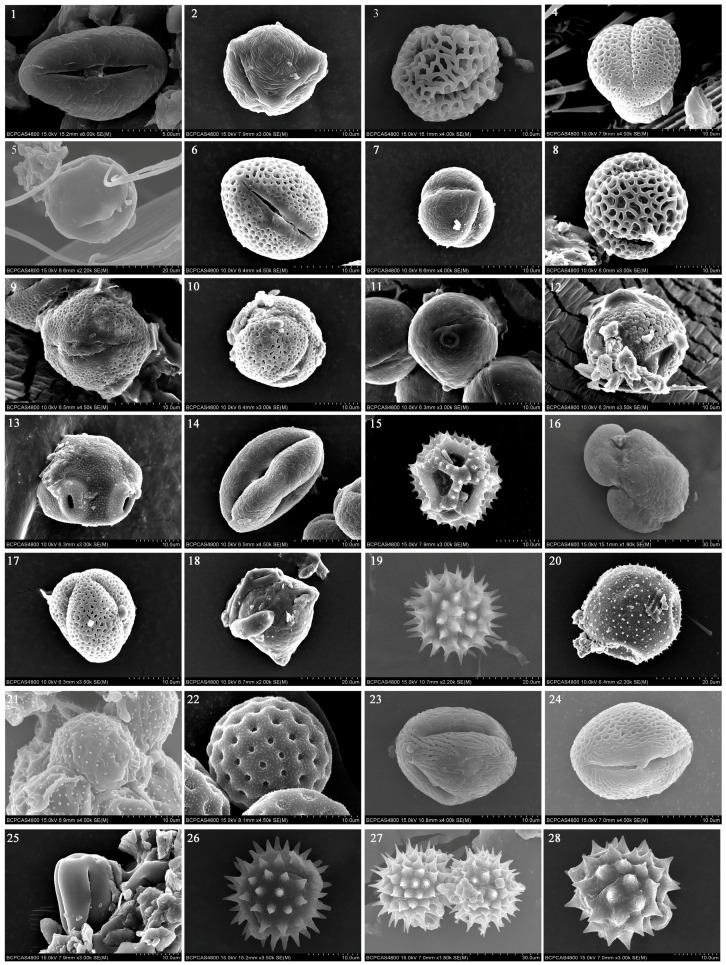
Scanning electron microscopy (SEM) microphotographs of the examined pollen species. 1. *Castanea mollissima*; 2. *Prunus yedoensis/Prunus subhirtella/Prunus serrulata*; 3. *Ligustrum lucidum/Ligustrum sempervirens*; 4. *Citrus sinensis*; 5. *Melia azedarach*; 6. *Flueggea virosa*; 7. *Hoboellia parviflora/Hoboellia grandiflora*; 8. *Olea europaea*; *9. Amorpha fruticosa*; 10. *Alniphyllum fortunei*; 11. *Robinia pseudoacacia*; 12. *Llex cornuta/Ilex corallina*; 13. *Pterocarya rhoifolia*; 14. *Castanopsis echinocarpa*; 15. *Taraxacum officinale/Taraxacum platypecidium*; 16. *Pinus tabuliformis/Pinus thunbergii/Pinus nigra/Pinus densiflora/Pinus kesiya/Pinus sylvestris/Pinus tropicalis*; 17. *Brassica rapa/Brassica napus/Brassica oleracea/Brassica juncea*; 18. *Elaeagnus umbellata*; 19. *Helianthus annuus*; 20. *Adenophora trachelioides/Adenophora remotiflora*; 21*. Smilax* L.; 22. *Chenopodium album*; 23*.* Rosaceae; 24. Rosaceae; 25. Violaceae (close to *Viola*); 26. Compositae (close to Coreopsis, *Sigebeckia*); 27. Compositae (also similar to *Cosmos*, *Kalimeris*, *Aster*); 28. *Aster* L. (also similar to *Chrysanthemum*, *Matricaria*).

Twenty-eight pollen species from at least 18 families were detected on *A. ipsilon* moths. The combination of DNA data using *rbcL*, pollen morphology, and distribution data of plant allowed us to identify 12 samples to the species level, including *Castanea mollissima* Blume, *Citrus sinensis* Blanco, *Melia azedarach* Linn., *Flueggea virosa* (Roxb. Ex Woigt) Voigt, *Olea europaea* L., *Amorpha fruticosa* Linn*.*, *Alniphyllum fortunei* (Hemsl.) Makino, *Robinia pseudoacacia* L., *Pterocarya rhoifolia* Siebold et Zucc., *Castanopsis echinocarpa* Miq., *Elaeagnus umbellata* Thunb. and *Chenopodium album* L., and 11 of the samples to the genus level, including *Prunus*, *Ligustrum*, *Holboellia*, *Llex*, *Taraxacum*, *Pinus*, *Helianthus*, *Brassica*, *Adenophora*, *Smilax*, and *Aster*. (Table 1, Figure 1, Supplementary Text S1). The success rates for a combination of pollen morphology, DNA barcoding, distribution data to the species-level and genus-level identification were 42.86% and 82.14%, respectively, while DNA barcoding were 21.43% and 78.57%, and pollen morphology were 7.14% and 71.43%, respectively. 

### 2.2. Frequency of Pollen Detection Rates on Body Parts of A. ipsilon

**Figure 2:**
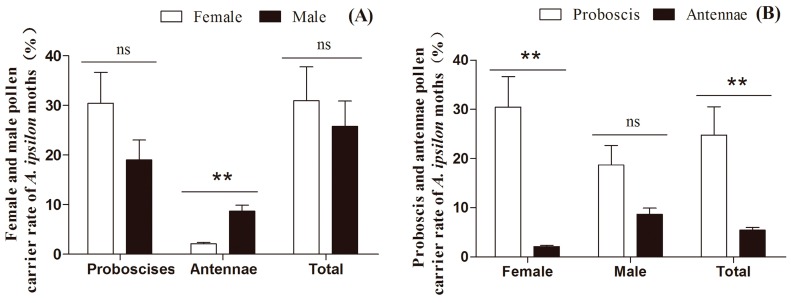
(**A**) Frequencies of pollen deposition on female and male proboscises, antennae and total (proboscis and antennae) of *A. ipsilon* moths; (**B**) Frequencies of pollen deposition on proboscis and antennae of female, male and total (female and male) of *A. ipsilon* moths. Double asterisks (**) indicates there was significant difference at the 1% level as determined by a *t*-test, and ns indicates there was no significant difference.

For whole body analyses, there was no significant sex-related difference in frequency of pollen occurrence on *A. ipsilon* moths, either for year groups (2012–2014) (Table 2, Figure 2), with 30.9% of female and 25.8% of male moths contaminated with plant pollens, or years analyzed individually: 2012, 2013 and 2014 (Table 2, Supplementary Figure S1C).

For the proboscises only, pollen detection rates were higher on female moths (30.4%) than males (19.0%) for 2012–2014 as a group (Table 2, Figure 2), and significantly for the same years individually: 2012, 2013 and 2014 (Table 2, Supplementary Figure S1A).

However, the frequency of pollen occurrence on antennae was lower for females (2.1%) than for males (8.7%) during 2012–2014 as a group (Table 2, Figure 2), and for the same years individually: 2012, 2013 and 2014 (Table 2, Supplementary Figure S1B).

The frequency of pollen deposits was higher on the proboscis (24.7%) than the antennae (5.4%), for 2012–2014 as a group (Table 2, Figure 2), and for the same years individually: 2012, 2013 and 2014 (Table 2, Supplementary Figure S1F). This difference was true for both female moths (Table 2, Supplementary Figure S1D,E).

**Figure 3:**
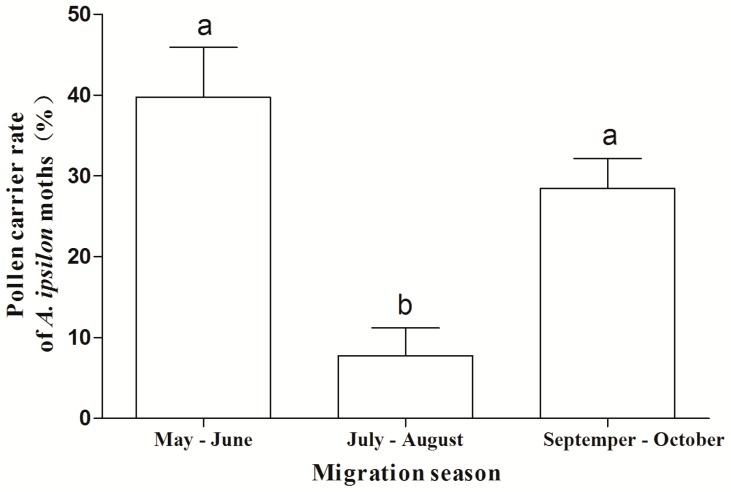
Frequencies of pollen deposition on migratory *A. ipsilon* near the Bohai Sea area in different migration stages during 2012–2014. Bars sharing the same letter mean there were no significant differences at the 5% level by Tukey’s honestly significant difference (HSD) tests.

With respect to the phenology of migration, the frequency of pollen deposits on bodies of *A. ipsilon* in the early part of the migration period (39.7%) and in the late period (28.5%) were both significantly higher than that in the middle period of the migration season (7.8%) during 2012–2014 (*F* = 12.61, *df*_1_ = 2, *df*_2_ = 6, *p* = 0.007) (Figure 3).

### 2.3. Characteristic of Plants Whose Pollen Was Found on A. ipsilon

**Figure 4:**
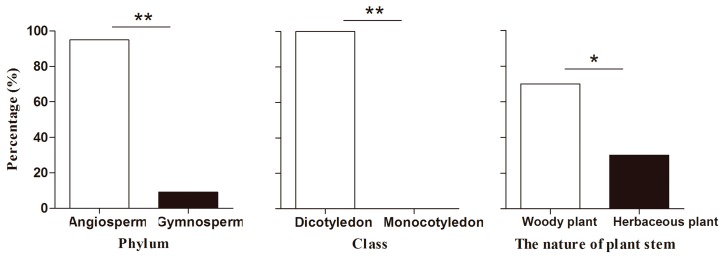
The characteristic of pollen source plants of migratory *A. ipsilon* during 2012–2014. Single asterisk (*) or double asterisks (**) indicates there was significant difference at the 5% or 1% level as determined by chi-squared test.

Chi-squared tests showed that significantly more species whose pollen was found on *A. ipsilon* moths were Angiosperm and Dicotyledon plants than Gymnosperms (*χ*^2^ = 32.40, *df* = 1, *p* < 0.001) or Monocotyledons (*χ*^2^ = 40.00, *df* = 1, *p* < 0.001). This led us to determine that moths visited woody plants more often than herbaceous plants (*χ*^2^ = 6.40, *df* = 1, *p* = 0.011) (Figure 4).

## 3. Discussion

We identified host plant used by *A. ipsilon* moths using DNA bar coding, pollen morphology, and known distributions of plants. Our results indicated that *A. ipsilon* moths fed on nectar from a very wide of plants, including 28 species from 18 families of mainly Dicotyledoneae in the Angiosperms. Kishimoto-Yamada *et al.* (2013) inferred host plant families of chrysomelid beetles including herbs, shrubs and vines [27]. However, a preference of *A. ipsilon* for visiting woody plants more than herbaceous plants was detected in our study. Different host plants can play important roles in the population increase of insects [28]. Both quality and quantity of food influence insect development and fecundity [29], and fitness effects may differ between different sources of nectar. Therefore, further research is needed to explore the preference of *A. ipsilon* moths for particular host plants.

*Agrotis ipsilon* is a long-distance migratory pest, often undertaking trans-regional migrations. The identification of pollen species can be used to determine the geographical origin of the insect since some plants grow only in certain ecological zones or geographic locations. For example, Hendrix and Showers (1992) found that *A. ipsilon* adults captured in Iowa and Missouri contained pollen from plants that only grow in south and southwest Texas [4]. Our results found pollen from *Ligustrum lucidum* Ait.*/Ligustrum sempervirens* (Franch.) Lingelsh, *C. sinensis*, *M. azedarach*, *Hoboellia parviflora* (Hemsl.) Gagnepl.*/Hoboellia grandiflora* Réaub., *O. europaea*, *A. fruticosa*, *A. fortunei*, *R. pseudoacacia*, *llex cornuta* Lindl. Et Paxton*/Ilex coralline* Franch. and *C. echinocarpa* on *A. ipsilon* adults captured in Beihuang from late May to June. Since these species grow in the Yangtze River basin and further south (over 800 km) [29], these adults must have foraged on these flowers there, then migrated northward to Beihuang, providing evidence for the northward spring migration of *A. ipsilon* [22].

Our use of DNA-based pollen analysis to provide species-level plant identifications validates this novel method, which has great potential for the study of plant-herbivore interactions [15]. The Consortium for the Barcode of Life-plant Working Group (CBOL) has recommended the two-locus combination of *rbcL* + *matK* as the best plant barcode due to its universality, sequence quality, and species discrimination [30]. Unfortunately, *matK* is difficult to amplify universally using currently available primer sets [31]. We chose the *rbcL* intron mainly because it has the highest level of coverage in GenBank among potential barcoding markers; the universality of PCR primers. A recent study also demonstrated that the *rbcL* sequences successfully identified the host plants families of rolled-leaf beetles [16]. *rbcl* is a good DNA barcoding region for plants at the family and genus levels, but for identification at the species level, a combination of pollen morphology and DNA barcoding is required. The efficiency of traditional pollen identification, which is highly dependent on human expertise and prohibitively time-consuming for large-scale studies, is improved by the use of DNA barcoding.

Our results showed that the frequency of pollen deposits on the proboscis was higher on female than male moths, which may be due to differences in the nutrient requirements of the sexes, with females generally having a higher nutrient demand than males [32]. The reason for higher frequencies of pollen deposits on antennae of male *vs.* female moths may be due to the different shape of the antennae between the sexes. The more branched antennae of male *A. ipsilon* moths are more favorable for pollen retention due to their greater surface area.

For the proboscis, when pollen was found, many grains were typically present, suggesting active contact through feeding rather than casual contact through wind-blown contamination. Pollen were rarely found on the antenna (5.4%), so future studies should focus on examining pollen found on *A. ipsilon* proboscises. Differences in the frequency of pollen deposits on *A. ipsilon* throughout the migratory period may be caused by differences in the abundance of nectar plants or the nutritional requirements of the moths, a topic that requires further investigation.

Flowers and insects have co-evolved [8], and the relationship between flowering plants and pollinators can be seen as mutually beneficial. Adult herbivores require nutrients or energy supplements from flowers for reproduction or flight, and flower-visiting herbivores are important pollinators [33]. The yield of many crops are dependent on the pollinators that visit their flowers, and yields are usually increased with the help of pollinators [4]. Our results showed *A. ipsilon* adults to be effective pollinators of *C. mollissima*, *C. sinensis*, *M. azedarach*, *F. virosa*, *O. europaea* and others. *A. ipsilon* is a long-distance disperser, undertaking regular migration across different agricultural areas. Therefore, the gene exchange of plants can be achieved across large regions with the help of *A. ipsilon* adults [34]. However, *A. ipsilon* larvae are pests of many crops, such as cotton, maize, potatoes, beans, and cruciferous vegetables [16]. Ultimately, the more knowledge we have about *A. ipsilon*, the better and more efficient the management practices that can be developed.

## 4. Experimental Section

### 4.1. Agrotis ipsilon Sampling

*Agrotis ipsilon* moths were collected using light traps. Moths were collected from sunset to sunrise, except during power outages or periods of heavy rain, using a vertical-pointing searchlight trap (model DK.Z.J1000B/t, 65.2 cm in diameter, 70.6 cm in height, and 30 in spread angle), equipped with a 1000-W metal halide-lamp (model JLZ1000BT; Shanghai Yaming Lighting Co., Ltd., Shanghai, China) on the top of a house (500 m elevation) on the 2.5 km^2^ island of Beihuang (BH, 38°24′ N; 120°55′ E) in the Bohai Gulf 40 km from the mainland to the north and 60 km from land to the south [34,35,36]. Collection were made every night from April to October during 2012–2014. Samples were collected with nylon net bags (60 mesh) which were changed manually every 2 h each night. Twenty moths (or all individuals if the total captured was <20) were removed from collection bags every morning. Each moth was killed by crushing its thorax, and it was placed into a 2 mL crytube and held at −20 °C in a freezer before microscopic examination.

### 4.2. Pollen Examination and Scanning Electron Microscopy (SEM) Preparation

Because Bryant *et al.* [37] found only 4% of pollen on *Helicoverpa zea* legs and eyes and suggested focusing examination efforts on the proboscis, in this study we examined the proboscis and antenna. *A. ipsilon* adult heads were removed from the body and examined at 200× magnification to make a preliminary identification of pollen adhering to their proboscis or antenna. To prevent contamination, a section of paper towel (9 × 9 cm) was placed on the microscope stage and changed after each sample, and forceps were also cleaned after each sample. Pollen grains found on a proboscis or antenna were mounted on aluminum stubs coated with gold in a sputter coater and immediately photographed under a Hitachi S-4800 cold field emission scanning electron microscope (Hitachi High-Technologies Co., Tokyo, Japan).

### 4.3. DNA Extraction from a Single Pollen Grain, PCR Amplification, Cloning and DNA Sequencing

Single pollen grains were isolated from the aluminum stubs and placed in individual PCR tubes containing 1 μL of lysis solution (NaOH with a concentration of 0.1 M, plus 2% Tween-20). NaOH and Tween-20 were purchased from the Beijing Chemical Reagent Co., Ltd. (Beijing, China). After the pollen grains were deposited, 5 μL of mineral oil was added. The samples were spun briefly (1 min at 1000× *g*) before incubation at 95 °C for 17 min 30 s to lyse the pollen grains. After lysis, equimolar aliquots of 1 μL Tris-EDTA (TE) buffer were added to neutralize the samples, which were then spun briefly [19]. The resulting solution was used as the template for Whole Genome Amplification (WGA) by illustra GenomiPhi V2 DNA Amplification Kit (GE Healthcare UK Ltd., London, UK). The WGA products of single pollen grains were used to amplify plant plastid DNA.

The partial region of chloroplast *rbcL* was amplified using PCR primers, namely *rbcla* forward (5′-ATGTCACCACAAACAGAAAC-3′), *rbcLa* reverse (5′-TCGCATGTACCTGCAGTAGC-3′) [38], *rbclb* forward (5′-ATGTCACCACAAACAGAAAC-3′) and *rbcLb* reverse (5′-GAAACGGTCTCTCCAACGCAT-3′) [39]. PCR amplification was performed using a thermal cycler (GeneAmp PCR System 9700, Applied Biosystems, Foster City, CA, USA) under the following conditions: initial activation at 94 °C for 4 min, 35 cycles of denaturation at 94 °C for 30 s, annealing at 55 °C for 30 s, and extension at 72 °C for 45 s, followed by final incubation at 72 °C for 10 min. Five sequencing replicates were taken for each pollen. The PCR reactions contained 100 ng of extracted DNA, 0.2 μM primer pairs, 2 mM dNTPs, 5 μL 10 × Long and Accurate (LA) Taq buffer, and 1 U LA Taq polymerase (TaKaRa, Beijing, China) for a total volume of 50 μL.

PCR products were purified with a Gel Extraction Kit (Tiangen, Beijing, China) and ligated directly into the pGEM-T Cloning Vector (Promega, Madison, WI, USA). Each DNA-containing plasmid was isolated from cultured *E. coli* cells by an alkaline miniprep method. The presence of inserts was verified by PCR with the M_13_ forward (5′-GTTTTCCCAGTCACGAC-3′) and reverse primers (5′-CAGGAAACAGCTATGAC-3′) and sequenced using Sanger sequencing by the company Biomed (Beijing, China).

### 4.4. Pollen Identification and the Characteristics of Pollen Source Plants

Identification of *rbcL* sequences was performed individually through similarity Basic Local Alignment Search Tool (BLAST) searches against GenBank [40]. The unknown sequence was considered a member of the best hit of the query sequences when it was completely consistent with them, and the unknown species sequence was considered to be the same genus as indicated by its top hits if there were differences between the sequences. Then the species were corrected according to their morphologic features and the known plant distributions. The pollen’s morphological features were identified using the published scanning electron microscopy (SEM) images in the atlas of pollen flora of China and pollen flora of China woody plants by SEM [41,42]. The pollen grains that could be classified to genus or species level were used to identify the source plants of pollen.

### 4.5. Data Analysis

Statistical analyses were conducted using SPSS 13.0 (SPSS Inc., Chicago, IL, USA) [43]. The frequency of pollen deposits on *A. ipsilon* during different migration phases was analyzed using one-way ANOVA, and the differences between the mean values were compared using Tukey’s HSD (honestly significant difference) test. The differences in the annual mean frequencies of pollen occurrence on female and male body parts (proboscis, antennae, or total (combined proboscis and antennae)) of *A. ipsilon* moths, and the differences in the annual mean frequency of pollen deposition on the proboscis or antennae of female, male, and total (female and male) *A. ipsilon* moths were analyzed by Student’s *t*-tests. The differences in frequency of pollen deposition on female and male body parts (proboscis, antennae or total (proboscis and antennae)) of *A. ipsilon* moths in each year, the differences of in such rates between proboscis and antennae of female, male and total (female and male) *A. ipsilon* moths in each year, and the characteristics of pollen source plants were all analyzed by a Chi-squared tests. All proportion data were logit transformed before being analyzed.

## 5. Conclusions

This study developed an effective approach for examining noctuid moth–host plant interactions. Unlike larvae that mainly feed on herbaceous plants, *A. ipsilon* moths obtain nutrition primarily from woody plants. We also found evidence that female *A. ipsilon* moths have a higher nutrient demand than males. Our results are helpful for understanding the coevolution processes between insects and their host plants, and for developing more efficient management practices of *A. ipsilon*.

## Figures and Tables

**Table 1:** Molecular and morphological identification of plant species by identifying pollen.

Number	Identified Plants	*rbcL*-Molecular Identification	Pollen Morphology Identification	Distributed in China
1	*Castanea mollissima*	Sister to *Castanea mollissima*/*Castanea sativa*	*Castanea* Mill./ *Castanopsis* Spach.*/Lithocarpus* Bl.	*Castanea mollissima*
2	*Prunus yedoensis/* *Prunus subhirtella/* *Prunus serrulata*	Sister to *Prunus yedoensis/* *Prunus subhirtella/Prunus serrulata*	*Prunus* L.	*Prunus yedoensis/* *Prunus subhirtella/* *Prunus serrulata*
3	*Ligustrum lucidum/* *Ligustrum sempervirens*	*Ligustrum lucidum/Ligustrum sempervirens*	Oleaceae	*Ligustrum lucidum/* *Ligustrum sempervirens*
4	*Citrus sinensis*	Sister to *Citrus maxima/Citrus sinensis*	*Citrus sinensis*	*Citrus maxima/Citrus sinensis*
5	*Melia azedarach*	Sister to *Melia azedarach*	*Melia* L.	*Melia azedarach*
6	*Flueggea virosa*	Sister to *Flueggea virosa/Flueggea neowawraea*	Eucommiaceae	*Flueggea virosa*
7	*Hoboellia parviflora/* *Hoboellia grandiflora*	Sister to *Stauntonia duclouxii/* *Hoboellia parviflora/Hoboellia grandiflora/* *Parvatia brunoniana*	*Holboellia* Wall.	*Stauntonia duclouxii/* *Hoboellia parviflora/* *Hoboellia grandiflora/* *Parvatia brunoniana*
8	*Olea europaea*	Sister to *Olea europaea*	Oleaceae	*Olea europaea*
9	*Amorpha fruticosa*	Sister to *Amorpha fruticosa/Amorpha canescens*	*Amorpha* L.	*Amorpha fruticosa*
10	*Alniphyllum fortunei*	Sister to *Alniphyllum fortunei*	*Alniphyllum fortunei*	*Alniphyllum fortunei*
11	*Robinia pseudoacacia*	Sister to *Robinia pseudoacacia*	*Robinia* L.	*Robinia pseudoacacia*
12	*Llex cornuta/Ilex corallina*	Sister to *Llex cornuta/Ilex corallina*	*Llex* L.	*Llex cornuta/Ilex corallina*
13	*Pterocarya rhoifolia*	Sister to *Pterocarya rhoifolia*	*Pterocarya* Kunth.	*Pterocarya rhoifolia*
14	*Castanopsis echinocarpa*	Sister to *Castanopsis echinocarpa*	*Castanea* Mill.*/Castanopsis* Spach./ *Lithocarpus* Bl.	*Castanopsis echinocarpa*
15	*Taraxacum officinale/* *Taraxacum platypecidium*	Sister to *Taraxacum alpinum/* *Taraxacum officinale/Taraxacum platypecidium*	*Taraxacum* Weber	*Taraxacum officinale/* *Taraxacum platypecidium*
16	*Pinus tabuliformis/* *Pinus thunbergii/Pinus nigra/* *Pinus densiflora/Pinus kesiya/* *Pinus sylvestris/Pinus tropicalis*	Sister to *Pinus tabuliformis/Pinus thunbergii/* *Pinus nigra/Pinus densiflora/Pinus densata/* *Pinus hwangshanensis/Pinus kesiya/* *Pinus sylvestris/Pinus tropicalis/* *Pinus yunnanensis*	*Pinus tabuliformis/Pinus thunbergii/* *Pinus nigra/Pinus densiflora/Pinus kesiya/* *Pinus sylvestris/Pinus tropicalis*	*Pinus tabuliformis/* *Pinus thunbergii/* *Pinus nigra/Pinus densiflora/* *Pinus kesiya/Pinus sylvestris/* *Pinus tropicalis*
17	*Brassica rapa/Brassica napus/* *Brassica oleracea/Brassica juncea*	Sister to *Brassica rapa/Brassica napus/* *Brassica oleracea/Brassica juncea*	Cruciferae	*Brassica rapa/Brassica napus/* *Brassica oleracea/* *Brassica juncea*
18	*Elaeagnus umbellata*	Sister to *Elaeagnus umbellata*	*Elaeagnus* L.	*Elaeagnus umbellata*
19	*Helianthus* L.	Sister to *Helianthus* L.	Compositae	*Helianthus* L.
20	*Adenophora trachelioides/* *Adenophora remotiflora*	Sister to *Adenophora trachelioides/* *Adenophora remotiflora/Hanabusaya asiatica*	*Adenophora* Flash.	*Adenophora trachelioides/* *Adenophora remotiflora*
21	*Smilax* L.	Sister to *Smilax* L.	*Smilax* L.	*Smilax* L.
22	*Chenopodium album*	Sister to *Chenopodium ficifolium/* *Chenopodium album*	*Chenopodium album*	*Chenopodium ficifolium/* *Chenopodium album*
23	Rosaceae	Unidentifiable	Rosaceae	Rosaceae
24	Rosaceae	Unidentifiable	Rosaceae	Rosaceae
25	Violaceae	Unidentifiable	Violaceae (close to *Viola*)	Violaceae
26	Compositae	Unidentifiable	Compositae (close to Coreopsis, *Sigebeckia*)	Compositae
27	Compositae	Unidentifiable	Compositae (also similar to *Cosmos*, *Kalimeris*, *Aster*)	Compositae
28	*Aster* L.	Unidentifiable	*Aster* L. (also similar to *Chrysanthemum*, *Matricaria*)	*Aster* L.

**Table 2:** Chi-squared test and a Student’s *t*-test for tested frequencies of pollen deposition on *A. ipsilon.*

Female and Male Pollen Carrier Rate of *A. ipsilon* Moths				Proboscis and Antennae Pollen Carrier Rate of *A. ipsilon* Moths			
Proboscis	2012	*χ*^2^	4.30	Female	2012	*χ*^2^	52.46
		*df*	1			*df*	1
		*p*	0.038			*p*	<0.001
	2013	*χ*^2^	8.56		2013	*χ*^2^	34.09
		*df*	1			*df*	1
		*p*	0.003			*p*	<0.001
	2014	*χ*^2^	10.57		2014	*χ*^2^	97.75
		*df*	1			*df*	1
		*p*	0.001			*p*	<0.001
	2012–2014	*t*	1.57		2012–2014	*t*	9.25
		*df*	4			*df*	4
		*p*	0.19			*p*	0.001
Antennae	2012	*χ*^2^	13.11	Male	2012	*χ*^2^	8.52
		*df*	1			*df*	1
		*p*	<0.001			*p*	0.004
	2013	*χ*^2^	4.39		2013	*χ*^2^	4.84
		*df*	1			*df*	1
		*p*	0.036			*p*	0.028
	2014	*χ*^2^	8.89		2014	*χ*^2^	11.34
		*df*	1			*df*	1
		*p*	0.003			*p*	0.001
	2012–2014	*t*	−6.73		2012–2014	*t*	2.62
		*df*	4			*df*	4
		*p*	0.003			*p*	0.059
Proboscis and antennae	2012	*χ*^2^	0.049	Female and male	2012	*χ*^2^	48.560
		*df*	1			*df*	1
		*p*	0.82			*p*	<0.001
	2013	*χ*^2^	2.10		2013	*χ*^2^	31.54
		*df*	1			*df*	1
		*p*	0.15			*p*	<0.001
	2014	*χ*^2^	3.74		2014	*χ*^2^	3.74
		*df*	1			*df*	1
		*p*	0.053			*p*	<0.001
	2012–2014	*t*	0.59		2012–2014	*t*	5.09
		*df*	4			*df*	4
		*p*	0.59			*p*	0.007

## Supplementary Materials

Supplementary materials can be found at http://www.mdpi.com/1422-0067/17/6/851/s1, Figure S1: Frequencies of pollen deposition on female and male proboscises (A), antennae (B) and total (proboscis and antennae) (C) of *A. ipsilon* moths; Frequencies of pollen deposition on proboscis and antennae of female (D), male (E) and total (female and male) (F) of *A. ipsilon* moths. Single asterisk (*) or double asterisks (**) indicates there was significant difference at the 5% or 1% level as determined by chi-squared test, and ns indicates there was no significant difference, Text S1: The *rbcl* sequences of the examined pollen species.
