# Supplementary material for: Host Plants Identification for Adult Agrotis ipsilon, a Long-Distance Migratory Insect
Source: Int J Mol Sci. 2016 Jun 2;17(6):851. doi: 10.3390/ijms17060851 (PMC4926385; doi:10.3390/ijms17060851)
Supplement: Supplementary file 1 [file ijms-17-00851-s001.zip › ijms-130670-Supplementary Materials/ijms-130670-supplementary-to be published.pdf]

Supplementary Materials: Host Plants Identification for Adult *Agrotis ipsilon*, a Long–Distance Migratory Insect

Yongqiang Liu, Xiaowei Fu, Limi Mao, Zhenlong Xing and Kongming Wu

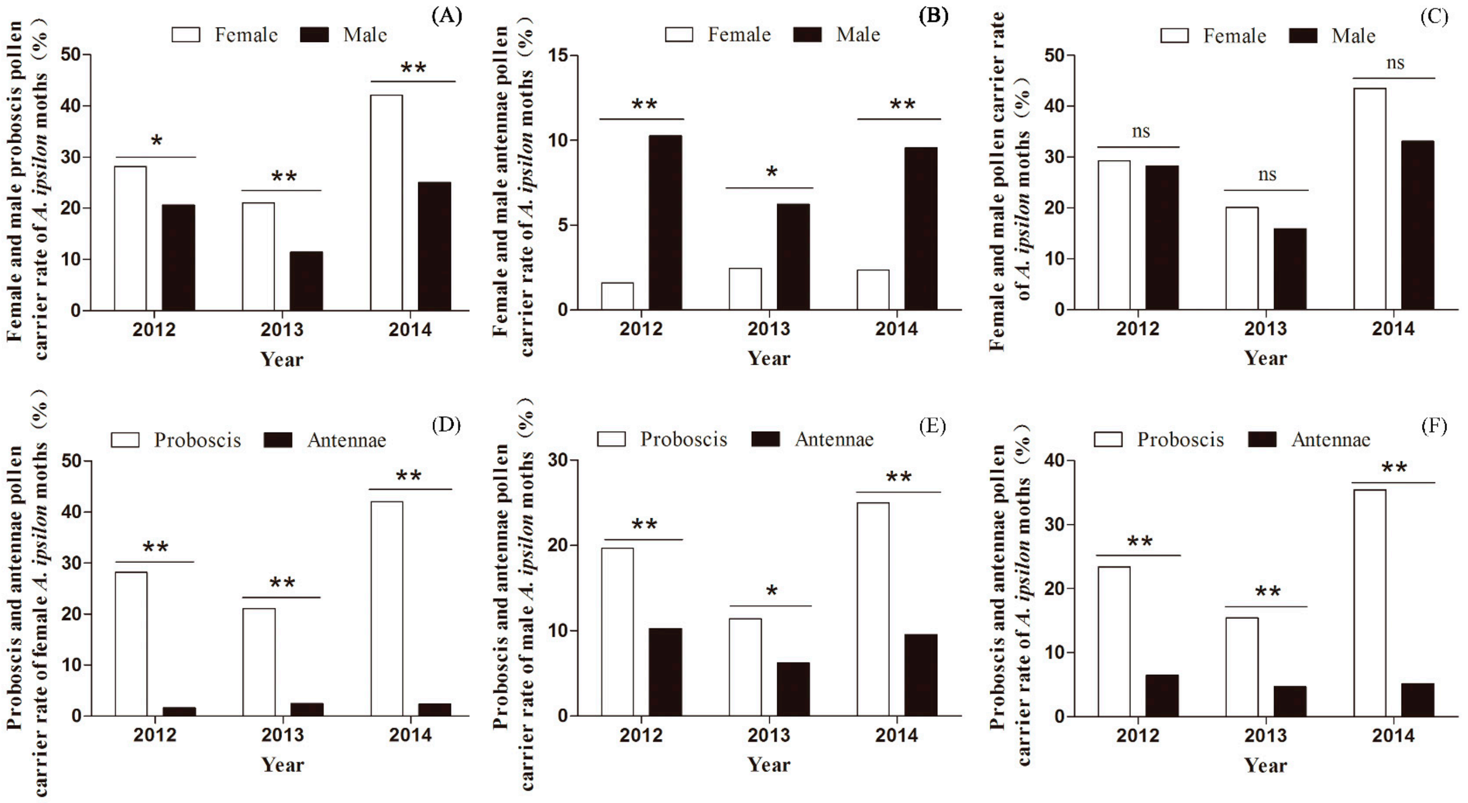

Figure S1. Frequencies of pollen deposition on female and male proboscises (**A**), antennae (**B**) and total (proboscis and antennae) (**C**) of *A. ipsilon* moths; Frequencies of pollen deposition on proboscis and antennae of female (**D**), male (**E**) and total (female and male) (**F**) of *A. ipsilon* moths. Single asterisk (*) or double asterisks (**) indicates there was significant difference at the 5% or 1% level as determined by chi-squared test, and ns indicates there was no significant difference.
